# Impact of COVID-19 on non-COVID intensive care unit service utilization, case mix and outcomes: A registry-based analysis from India

**DOI:** 10.12688/wellcomeopenres.16953.1

**Published:** 2021-06-18

**Authors:** Indian Registry of IntenSive care (IRIS), Neill KJ Adhikari, Abi Beane, Dedeepiya Devaprasad, Robert Fowler, Rashan Haniffa, Augustian James, Devachandran Jayakumar, Chamira Kodippily, Rohit Aravindakshan Kooloth, Rakesh Laxmappa, Kishore Mangal, Ashwin Mani, Meghena Mathew, Vrindha Pari, Sristi Patodia, Rajyabardhan Pattnaik, Dilanthi Priyadarshini, Mathew Pulicken, Ebenezer Rabindrarajan, Pratheema Ramachandran, Kavita Ramesh, Usha Rani, Ananth Ramaiyan, Nagarajan Ramakrishnan, Lakshmi Ranganathan, Aasiyah Rashan, Raymond Dominic Savio, Jaganathan Selva, Bharath Kumar Tirupakuzhi Vijayaraghavan, Swagata Tripathy, Ishara Udayanga, Ramesh Venkataraman

**Affiliations:** 1 Intedepartmental Division of Critical Care Medicine, University of Toronto, Toronto, Canada; 2 Mahidol Oxford Tropical Medicine Research Unit (MORU), Bangkok, Thailand; 3 Department of Critical Care Medicine, Apollo Specialty Hospital, Chennai, India; 4 Department of Critical Care Medicine, Apollo Main Hospital, Chennai, India; 5 Network for Improving Critical care Systems and Training, Colombo, Sri Lanka; 6 Department of Critical Care Medicine, Nanjappa Hospital, Shimoga, India; 7 Department of Critical Care Medicine, Eternal Hospital, Jaipur, India; 8 Department of Critical Care Medicine, Apollo First Med Hospital, Chennai, India; 9 Chennai Critical Care Consultants Private Limited, Chennai, India; 10 Department of Critical Care Medicine, Apollo Proton Cancer Centre, Chennai, India; 11 Department of Critical Care Medicine, Ispat General Hospital, Rourkela, India; 12 Department of Critical Care Medicine, Pushpagiri Medical College, Tiruvalla, India; 13 Department of Critical Care Medicine, ABC Hospital, Vishakapatnam, India; 14 Department of Critical Care Medicine, Mehta Hospital, Chennai, India; 15 Department of Anaesthesia and Intensive Care Medicine, All India Institute of Medical Sciences, Bhubaneswar, India

**Keywords:** COVID-19, registries, critical care, severity of illness

## Abstract

**Background:** Coronavirus disease 2019 (COVID-19) has been responsible for over 3.4 million deaths globally and over 25 million cases in India. As part of the response, India imposed a nation-wide lockdown and prioritized COVID-19 care in hospitals and intensive care units (ICUs). Leveraging data from the Indian Registry of IntenSive care, we sought to understand the impact of the COVID-19 pandemic on critical care service utilization, case-mix, and clinical outcomes in non-COVID ICUs.

**Methods:** We included all consecutive patients admitted between 1 ^st^ October 2019 and 27 ^th^ September 2020. Data were extracted from the registry database and included patients admitted to the non-COVID or general ICUs at each of the sites. Outcomes included measures of resource-availability, utilisation, case-mix, acuity, and demand for ICU beds. We used a Mann-Whitney test to compare the pre-pandemic period (October 2019 - February 2020) to the pandemic period (March-September 2020). In addition, we also compared the period of intense lockdown (March-May 31 ^st^ 2020) with the pre-pandemic period.

**Results:** There were 3424 patient encounters in the pre-pandemic period and 3524 encounters in the pandemic period. Comparing these periods, weekly admissions declined (median [Q1 Q3] 160 [145,168] to 113 [98.5,134]; p=0.00002); unit turnover declined (median [Q1 Q3] 12.1 [11.32,13] to 8.58 [7.24,10], p<0.00001), and APACHE II score increased (median [Q1 Q3] 19 [19,20] to 21 [20,22] ; p<0.00001). Unadjusted ICU mortality increased (9.3% to 11.7%, p=0.01519) and the length of ICU stay was similar (median [Q1 Q3] 2.11 [2, 2] vs. 2.24 [2, 3] days; p=0.15096).

**Conclusion:** Our registry-based analysis of the impact of COVID-19 on non-COVID critical care demonstrates significant disruptions to healthcare utilization during the pandemic and an increase in the severity of illness.

## Introduction

The coronavirus disease 2019 (COVID-19) pandemic has been responsible for over 3.4 million deaths globally as of 26 ^th^ May 2021 ( WHO COVID-19 Dashboard). In India, there have been over 25 million cases and approximately 300000 deaths ( WHO COVID-19 Dashboard for India). As part of the early pandemic response in the first wave, India and several other countries imposed nation-wide lockdowns and restrictions to control the spread of the disease ( The Hindu, BBC). Health-services were restructured with prioritization of COVID-19 care ( The Economic Times, The Times of India) including in hospitals and intensive care units (ICUs). The lockdown also disrupted public transport, limiting access to healthcare facilities. In addition, fears of contracting the infection dissuaded patients from seeking care for non-COVID-19 illnesses ( The Wire Science, TOI Plus).

In past smaller epidemics, health systems have struggled to maintain routine services and non-pandemic healthcare and public health services suffered ^1, 2^. During the ongoing COVID-19 pandemic, there is limited information on how the outbreak has impacted acute and critical care service provision in India and other low- and middle-income countries (LMICs).

Even in non-pandemic times, India has a limited supply of critical care capacity with bed availability estimated at 2.6 per 100,000 population compared with much higher capacity in most high-income countries, for example 12.9 ICU beds per 100,000 population in Canada ^3^, and other LMICs in the region, for example 11.7 beds per 100,000 population in Mongolia ^4^. Other well-described challenges to the delivery of critical care in India include the limited number of beds with capacity for oxygen delivery, ventilators, and importantly, healthcare professionals ^5^. All these challenges are further amplified by the disparities in distribution of these resources between urban and rural India ^6^ and between the public and private sector. 

In this context, critical care registries, by continually evaluating service provision, case-mix and outcomes, can be used to describe the impact of pandemic on critical care service provision and inform health-capacity strengthening efforts. The Indian Registry of IntenSive care (IRIS) prospectively collects information on service utilisation, case mix, and outcomes ^7^. Leveraging the registry platform, we sought to understand the impact of the COVID-19 pandemic on critical care service utilization, case-mix, severity of illness and clinical outcomes in non-COVID ICUs.

## Methods

### Data sources

All data for this analysis was available from IRIS. IRIS, a cloud-based registry established in January 2019, prospectively collects information on service utilisation, case-mix, and outcomes. 13 hospitals (13 ICUs) currently participate in the registry and details of implementation have previously been published ^7^.

For this report, we included all consecutive cases admitted between 1 ^st^ October 2019 and 27 ^th^ September 2020. Patients admitted before 27 ^th^ September 2020, but not discharged at time of analysis (2 ^nd^ November 2020) were excluded. Data was extracted from the registry database and included patients admitted to the non-COVID or general ICUs at each of the sites. In all participating hospitals, COVID-19 patients were managed in designated locations and as such, not included in the registry. No additional variables, other than those already available in the registry, were included in the study design.

### Variables

The main exposure was time, defined as the pre-pandemic period (October 2019-February 2020), pandemic period (March-September 2020), and period of intense lockdown (March-May 31 ^st^ 2020) within the pandemic period. Outcomes of interest included admission rates, unit occupancy, unit turnover, bed availability, use of resources at admission (invasive and non-invasive ventilation, vasoactive medications, renal replacement therapy), number of surgical admissions, route to admission (i.e. from operating room, emergency room, ward etc.) and case mix (diagnosis, APACHE II score ^8^).

Unit occupancy was defined in the registry as *weekly number of admissions **X** mean weekly length of stay/ number of beds available **X** 7.* Unit turnover was defined in the registry as *weekly number of discharges/ number of beds available **X** 7* and bed availability was defined as *(weekly number of admissions X mean length of stay)-(bed capacity X 7)*.

### Analysis

We used a Mann-Whitney test to compare baseline data from the pre-pandemic period to the pandemic period. In addition, we also compared the period of intense lockdown with the pre-pandemic period. October 2019 was chosen as the reference starting point as most units in the registry were contributing data by then (the registry was established in January 2019).

Data is presented as median (Q1, Q3) using weekly recorded numbers. We plotted 2020 data points with a loess smoothing line and standard error bars. Statistical analysis was conducted using RStudio v3.6.1 (R Foundation, Vienna, Austria). Full R code are provided (see *Code availability*) ^9^ and raw data are available with restrictions on access (see *Data availability*).

### Ethics and consent

This analysis has been approved by the Institutional Ethics Committee (Apollo Main Hospital-C-S-010/02-21) centrally at the study coordinating centre. The consent model for IRIS has been previously described ^7^. As this was a secondary analysis of deidentified registry data, no further individual patient-level consent was considered necessary or sought.

### Patient and public involvement

Given the nature of this analysis, no patient or public involvement was sought.

## Results

**Figure 1.**
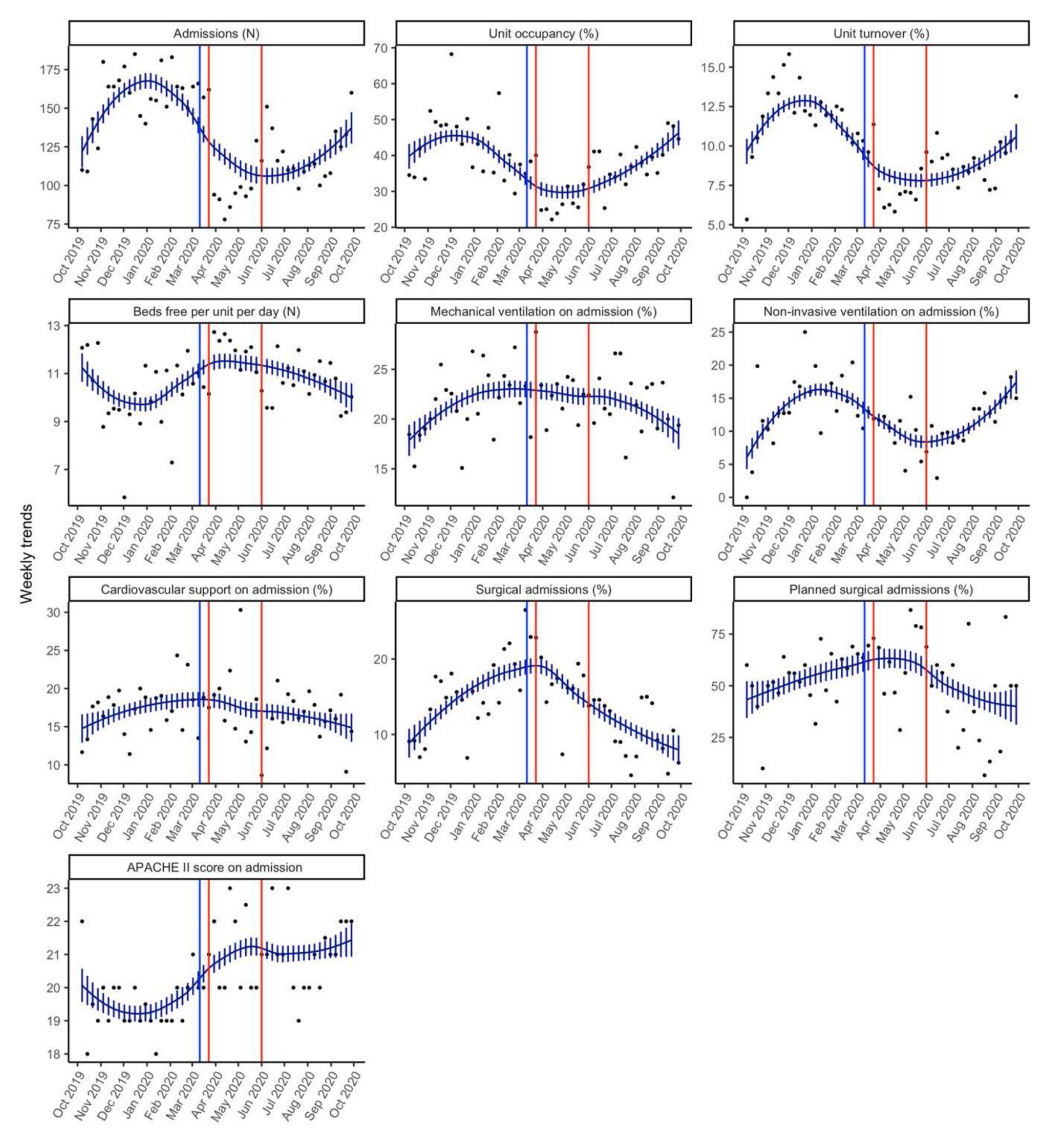
Smoothed weekly trends. The dots are the original data, and the lines and bars are loess predictions and standard errors. The blue line represents March 11 ^th^ 2020, the date the WHO declared Covid a pandemic. The brown lines indicate the intense lockdown period in India from 23 ^rd^ March 2020– 1 ^st^ June 2020.

**Figure 2.**
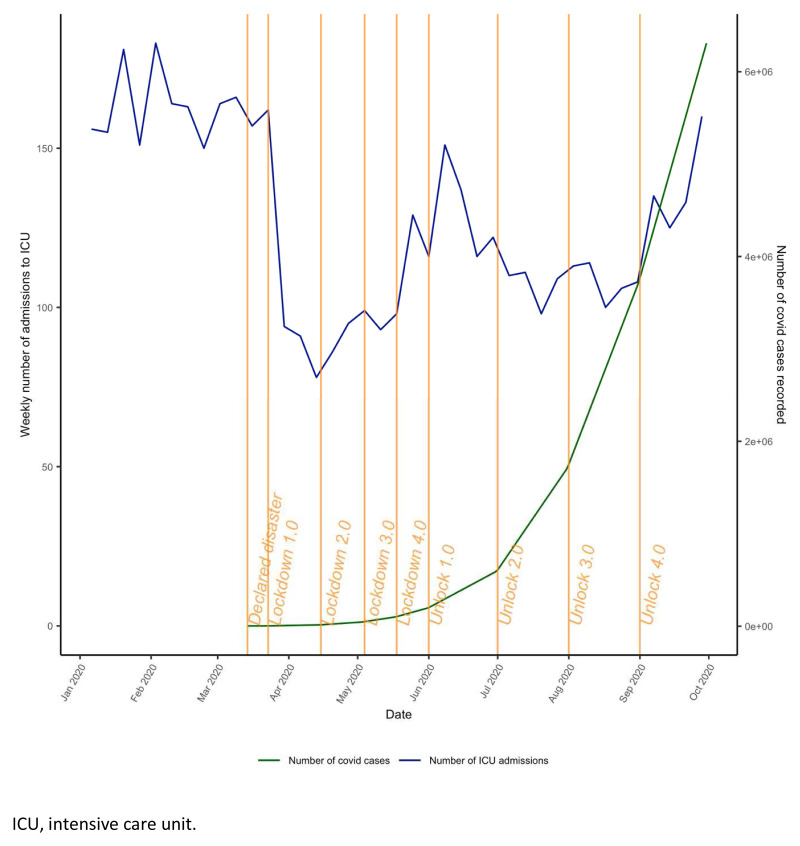
Phases of lockdown in India. ICU, intensive care unit.

A total of 6948 patient encounters from 13 hospitals (13 ICUs) were reported between 1 ^st^ October 2019 and 27 ^th^ September 2020, with 3424 encounters in the pre-pandemic period and 3524 encounters in the pandemic period ( Figure 1). All variables had <6% missing data. National lockdown commenced in India on the 24 ^th^ March 2020, and the first phase of unlocking started on 1 ^st^ June 2020 ( Figure 2).

### Number of admissions, case-mix and turnover

Weekly admissions declined from a median (Q1, Q3) of 160 (145, 168) admissions from the pre-pandemic period to 113 (98.5, 134.0) for the pandemic period (p=0.00002) ( Table 1). For this same comparison period there was also a significant decline in the unit turnover from a median (Q1, Q3) of 12.1% (11.3, 13.1) to 8.6% (7.2, 10.0) (p<0.00001) and unit occupancy from a median (Q1, Q3) of 43.2% (35.2, 49.0) to 35.1% (29.0, 40.0) ( Table 1).

**Table 1.** Comparison of pre and pandemic period. All variables are reported as weekly median (Q1, Q3). A Mann-Whitney test was performed to statistically test the difference between the two time periods.

Variable	Oct 2019 to Feb 2020	Mar to Sep 2020	p-value
Admissions (N)	160 (145, 168)	113 (98.5, 134)	**0.00002**
Unit occupancy (%)	43.19 (35.23, 49)	35.12 (29.01, 40)	**0.00347**
Unit turnover (%)	12.1 (11.32, 13)	8.58 (7.24, 10)	**<0.00001**
Beds free per unit per day (N)	10.13 (9.3, 11)	11.01 (10.35, 12)	**0.03425**
Mechanical ventilation on admission (%)	22.01 (19.01, 24)	22.37 (19.49, 24)	0.85204
Non-invasive ventilation on admission (%)	14.56 (11.57, 17)	11.43 (9.41, 13)	**0.01312**
Cardiovascular support on admission (%)	17.65 (14.57, 19)	16.98 (14.56, 19)	0.64773
Surgical admissions (%)	14.59 (12.18, 18)	14.29 (9.05, 16)	0.63438
Planned surgical admissions (%)	51.85 (46.43, 60)	56.25 (37.5, 69)	0.75095
APACHE II score on admission	19 (19, 20)	21 (20, 22)	**<0.00001**

In terms of case-mix, there were no differences in the proportion of surgical admissions between these periods.

Comparing the pre-pandemic phase to the intense lockdown phase (March-May 2020), the median (Q1, Q3) weekly admissions declined to 98 (93, 157) admissions (p=0.00306). In addition, for this comparison period, the percentage unit occupancy as well as unit turnover were also significantly lower during the intense lockdown period as compared to the pre-pandemic period ( Table 2). The number of beds free per day per week increased from a median (Q1, Q3) of 10.13 (9.3, 11) to 11.92 (11.01, 12) (p=0.00516) during the intense lockdown phase ( Table 2).

For this comparison period, the proportion of surgical admissions increased from a median (Q1, Q3) of 14.6% (12.18, 18) to 17.4% (16.13, 20).

### Severity of illness

The median APACHE II score (Q1, Q3) increased significantly from 19 (19, 20) in the pre-pandemic period to 21 (20, 22) during the pandemic period (p<0.00001) ( Table 1). However, the median percentage of patients needing mechanical ventilation at admission was not different (22.01 vs. 22.37; p=0.85). There was a decline in the median percentage of patients needing non-invasive ventilation at admission from 14.56 (11.57, 17) to 11.43 (9.41, 13) (p=0.013). Need for cardiovascular support at admission was not different between the two periods (17.65% vs. 16.98%; p=0.65) ( Table 1).

Comparing the pre-pandemic period to the intense lockdown phase, the median APACHE II was significantly higher (19 vs. 20) (p=0.0001) ( Table 2). The median proportion of patients needing mechanical ventilation was not different between these two periods ( Table 2). Similar to the previous comparison period, the median proportion of patients needing non-invasive ventilation was lower during the intense lockdown phase (14.56 vs. 11.58; p=0.02).

**Table 2.** Comparison of pre pandemic and intense lockdown periods. All variables are reported as median (Q1, Q3). A Mann-Whitney test was performed to statistically test the difference between the two time periods.

Variable	Oct 2019 to Feb 2020	Mar to May 2020	p-value
Admissions (N)	160 (145, 168)	98 (93, 157)	**0.00306**
Unit occupancy (%)	43.19 (35.23, 49)	26.66 (25.02, 35)	**0.00007**
Unit turnover (%)	12.1 (11.32, 13)	7.1 (6.59, 10)	**0.00008**
Beds free per unit per day (N)	10.13 (9.3, 11)	11.92 (11.01, 12)	**0.00516**
Mechanical ventilation on admission (%)	22.01 (19.01, 24)	22.48 (21.05, 24)	0.59499
Non-invasive ventilation on admission (%)	14.56 (11.57, 17)	11.58 (10.2, 12)	**0.02124**
Cardiovascular support on admission (%)	17.65 (14.57, 19)	18.52 (14.74, 19)	0.73635
Surgical admissions (%)	14.59 (12.18, 18)	17.44 (16.13, 20)	**0.02406**
Planned surgical admissions (%)	51.85 (46.43, 60)	65.38 (56.25, 73)	**0.02926**
APACHE II score on admission	19 (19, 20)	20 (20, 22)	**0.0001**

### Outcomes

**Figure 3.**
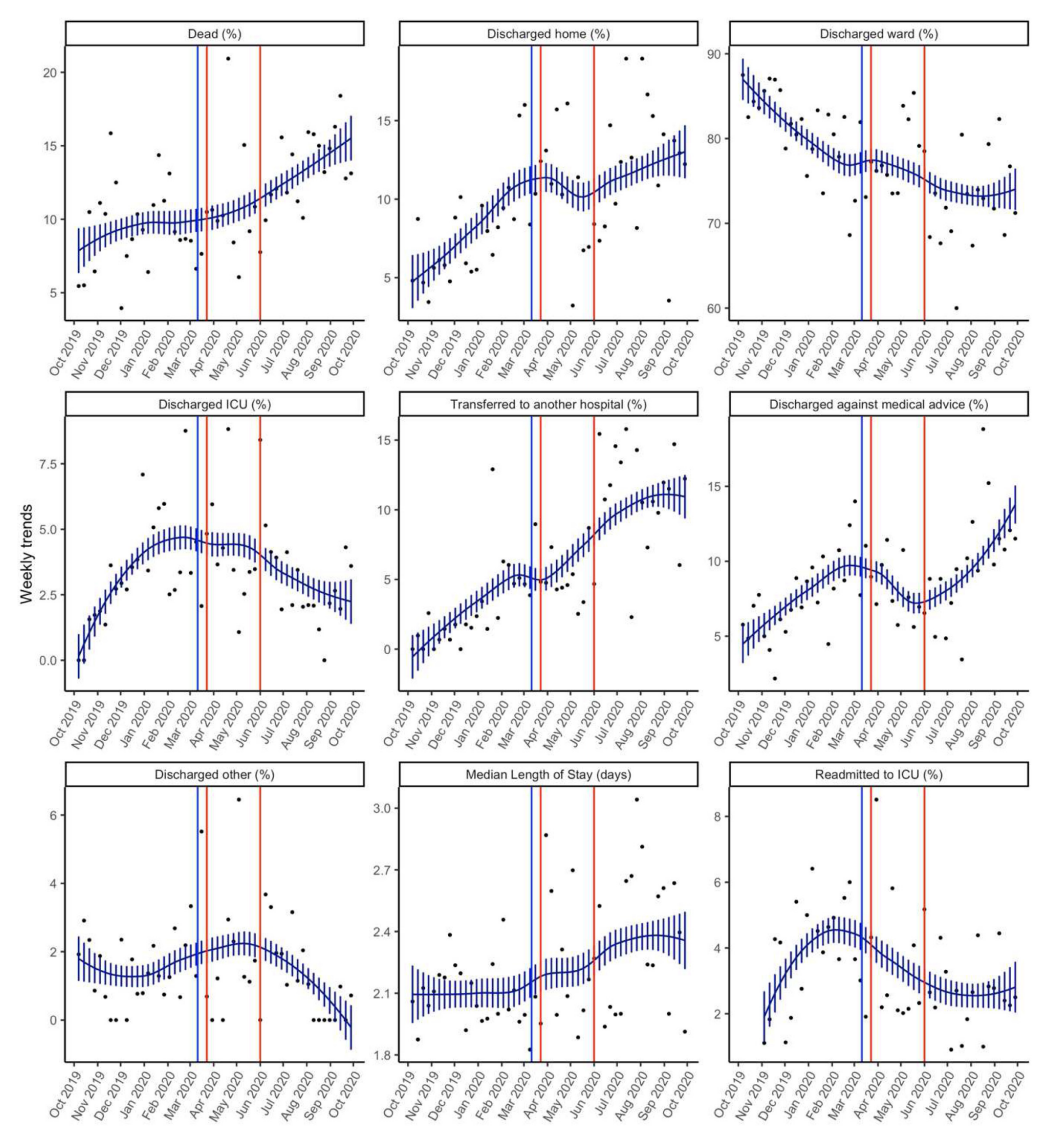
Smoothed weekly trends of outcomes. The dots are the original data, and the lines and bars are loess predictions and standard errors. The blue line represents March 11 ^th^ 2020, the date the WHO declared COVID-19 as a pandemic. The brown lines indicate the intense lockdown period in India from 23 ^rd^ March 2020– 1 ^st^ June 2020.

Table 3 and Figure 3 presents details of the key outcomes in the pandemic and pre-pandemic periods. There was a higher overall unadjusted ICU morality during the pandemic period as compared to the pre-pandemic period (11.7% vs 9.3%, p=0.01519). There was no difference in the median length of stay between the two periods.

**Table 3.** Key outcomes: pre-pandemic and pandemic period. All variables are reported as the weekly median with interquartile range. A Mann-Whitney test was performed to statistically test the difference between the two time periods.

Variable	Oct 2019 to Feb 2020	Mar to Sep 2020	p-value
Dead (%)	9.29 (7.5, 11)	11.68 (9.91, 15)	**0.01519**
Discharged home (%)	6.45 (5.51, 9)	12.23 (8.4, 14)	**0.00018**
Discharged ward (%)	82.52 (78.82, 84)	73.56 (71.79, 79)	**0.00003**
Discharged ICU (%)	2.94 (1.88, 4)	3.45 (2.09, 4)	0.70912
Transferred to another hospital (%)	1.76 (0.68, 3)	8.7 (4.67, 12)	**<0.00001**
Discharged against medical advice (%)	7.03 (5.29, 9)	9.38 (7.18, 11)	**0.01419**
Discharged other (%)	1.29 (0.75, 2)	1.15 (0, 2)	0.91036
Readmitted to ICU (%)	4.27 (2.76, 5)	2.65 (2.17, 4)	0.05106
Median length of stay (days)	2.11 (2, 2)	2.24 (2, 3)	0.15096

ICU, intensive care unit.

## Discussion

Our registry-based analysis of the impact of COVID-19 on non-COVID critical care services shows a significant reduction in the median number of admissions to the ICUs during the pandemic period as compared to the pre-pandemic period. This reduction was most prominent during the intense lockdown phase. We also noted an increase in the median APACHE II score and a significant decline in the ICU turnover for comparison periods. While the median severity of illness was higher, we did not see an increase in the proportion of patients needing mechanical ventilation or cardio-vascular support at admission. Additionally, the proportion of planned surgical admissions declined over this period but was not statistically significant. Unadjusted ICU mortality was higher during the pandemic period, but the median length of stay was similar.

To our knowledge, our study is the first to demonstrate the collateral impact of the pandemic on non-COVID acute and critical care. In previous epidemics, similar findings have been reported. However, the scale and the extended timeline of the current pandemic means that such disruption is likely of much a larger magnitude than previously witnessed. Lee and colleagues reported a 33% reduction in emergency room visits during the MERS-CoV outbreak in South Korea ^10^. Similarly, during the SARS outbreak, Huang and colleagues showed a mean reduction of 92.5±8.3 patients in emergency room visits ^11^.

Such disruptions to other components of healthcare delivery have been reported during previous outbreaks and epidemics such as the impact of Ebola on maternal and child health services in Guinea ^12^ and of SARS on ambulatory and in-patient care ^13^. During the current pandemic, concerns have been expressed about the impact on hematopoietic stem cell transplant ^14^, on outcomes from cancer surgery ^15^, on maternal and neonatal care ^16^, and tuberculosis control programs ^17, 18^.

There are several reasons for such disruptions, including the necessary and inevitable prioritization of pandemic healthcare services, obstacles to access of healthcare triggered by lockdown and suspension of public transport, fear of contracting infection whilst trying to access care, among others. Such medical avoidance behaviours have also been described during past outbreaks ^19^. In India, a nationwide lockdown was imposed on 24 ^th^ March 2020 with an immediate disruption of public transport and the imposition of a curfew across the country. Additionally, hospitals were explicitly asked to prioritize COVID-19 care by increasing capacity, by redirecting resources (manpower, equipment etc.), and reducing or suspending non-emergency services.

In a recent survey of healthcare worker perceptions on the impact of the pandemic led by our group ^20^, respondents reported a decline in acute care service utilisation and a delay in time-sensitive procedures such as percutaneous coronary angioplasties. In the view of the respondents, fear of contracting infection and lockdown were seen as having contributed the most to such disruptions. The results of the current study provide objective data to complement the perceptions of the survey respondents.

### Strengths and limitations

Our study has several important strengths. We compared the service utilisation during the pandemic and the intense lockdown phase with an extended pre-pandemic period to overcome any limitations arising from secular trends. Missingness for our dataset was less than 6% for all variables and the reported changes are therefore not due to systematic differences in data availability for the pre and pandemic periods. In addition, our study, to the best of our knowledge, is the first to provide objective data on disruptions to acute and critical care.

Our report has several limitations. Our network includes only a fraction of ICUs within India and may not be epidemiologically representative of the impact of the pandemic on critical care service utilisation across the country or wider region. The impact on ICU service utilisation described may be due to other agents external to the registry’s surveillance and are not described here. In addition, we did not include data from COVID ICUs nor did we compare the service utilization between the COVID and non-COVID areas. Mortality in our cohort is low (9.3 and 11.7%) as compared to other registries (for example 31% for the year 2019 in the Linking Of Global Intensive Care or LOGIC initiative) and this may be explained by a combination of reasons including the mix of ICUs represented in IRIS and the overall small number of ICUs currently participating. We are also unable to comment on the effect of the pandemic on population-level health indicators. Nonetheless, we think this report provides valuable insights into the impact of public health decision and policy making during the pandemic and the impact of the pandemic on healthcare resource utilisation.

## Conclusion

Our registry-based analysis of the impact of COVID-19 on non-COVID critical care demonstrates significant disruptions to healthcare utilization during the pandemic and an increase in the severity of illness. Our findings are likely to inform future healthcare planning during pandemics to minimise disruptions to other aspects of healthcare delivery.

## Data availability

### Underlying data

Pooled data from IRIS are available from the IRIS Dashboard at https://nicst.com/picu-iris-public/. The IRIS collaboration supports and welcomes data sharing. Our agreement with participating sites in the registry is only for the sharing of deidentified data between them and the registry coordinating centre for the purposes of audit, quality improvement and specific research questions. We are not allowed to post data on a repository or any other public database. However, raw data will be made available to qualified researchers who provide a detailed and methodologically sound proposal with specific aims that are clearly outlined. Such proposals will be screened by the IRIS Steering committee for approval.

Data sharing will be for the purposes of medical research and under the auspices of the consent under which the data were originally gathered. To gain access, qualified researchers will need to sign a data sharing and access agreement and will need to confirm that data will only be used for the agreed upon purpose for which data access was granted. Researchers can contact the corresponding author through electronic mail ( bharath@icuconsultants.com) for such access; alternatively, IRIS can be contacted at info@irisicuregistry.org and joinus@irisicuregistry.org.

## Code availability

Analysis code available from: https://github.com/NICST-PROTECT/ICU-service-utilisation-public/tree/v1.0.1

Archived analysis code at time of publication: https://doi.org/10.5281/zenodo.4939947 ^9^

License: MIT
