# Supplementary material for: Impact of COVID-19 on non-COVID intensive care unit service utilization, case mix and outcomes: A registry-based analysis from India
Source: Wellcome Open Res. 2021 Nov 10;6:159. Originally published 2021 Jun 18. [Version 2] doi: 10.12688/wellcomeopenres.16953.2 (PMC8666986; doi:10.12688/wellcomeopenres.16953.2)
Supplement: Peer review history

# Peer review: Reviewer response for version 2

**DOI:** 10.21956/wellcomeopenres.19216.r47013

**Published:** 2021-12-07

**Authors:** Kate Tatham

**Affiliations:** 1 Division of Anaesthetics, Pain Medicine & Intensive Care, Department of Surgery & Cancer, Faculty of Medicine, Imperial College London, London, UK

I approve this article.

 Minor comment- please state that normality testing was performed and that the data were non-parametric and hence Mann-Whitney U was utilised.

Is the work clearly and accurately presented and does it cite the current literature?

Yes

If applicable, is the statistical analysis and its interpretation appropriate?

I cannot comment. A qualified statistician is required.

Are all the source data underlying the results available to ensure full reproducibility?

Yes

Is the study design appropriate and is the work technically sound?

Yes

Are the conclusions drawn adequately supported by the results?

Yes

Are sufficient details of methods and analysis provided to allow replication by others?

Yes

Reviewer Expertise:

Intensive Care

I confirm that I have read this submission and believe that I have an appropriate level of expertise to confirm that it is of an acceptable scientific standard.

# Peer review: Reviewer response for version 2

**DOI:** 10.21956/wellcomeopenres.19216.r47014

**Published:** 2021-11-12

**Authors:** Saptharishi Lalgudi Ganesan

**Affiliations:** 1 Department of Paediatrics, Schulich School of Medicine and Dentistry, Western University, London, ON, Canada; 2 Children's Hospital of Western Ontario, London Health Sciences Center, London, ON, Canada; 3 Brain & Mind Institute, Western University, London, ON, Canada

I would like to thank the authors for considering my suggestions and providing clear responses to each comment. I have no further comments to make. The reviewer suggestions have been incorporated wherever feasible.

Is the work clearly and accurately presented and does it cite the current literature?

Yes

If applicable, is the statistical analysis and its interpretation appropriate?

Yes

Are all the source data underlying the results available to ensure full reproducibility?

Yes

Is the study design appropriate and is the work technically sound?

Yes

Are the conclusions drawn adequately supported by the results?

Yes

Are sufficient details of methods and analysis provided to allow replication by others?

Yes

Reviewer Expertise:

Critical Care, Global Health, Clinical epidemiology, Neurocritical Care, Child Health

I confirm that I have read this submission and believe that I have an appropriate level of expertise to confirm that it is of an acceptable scientific standard.

# Peer review: Reviewer response for version 1

**DOI:** 10.21956/wellcomeopenres.18709.r45550

**Published:** 2021-10-14

**Authors:** Kate Tatham

**Affiliations:** 1 Division of Anaesthetics, Pain Medicine & Intensive Care, Department of Surgery & Cancer, Faculty of Medicine, Imperial College London, London, UK

Thank you for asking me to review this manuscript analysing non-COVID ICU utilisation before and during the peak COVID pandemic in India. It demonstrates a significant disruption in healthcare utilisation during this time.

 Overall the study is well designed and well written and acknowledges its strengths and limitations.

**Major comments are:**

- Could you comment on how the time periods were assigned. Would it be better to compare equal time periods (currently 5 months versus 7 months)?
- Can you elaborate on how the unit occupancy equation definition was decided on? Did it include mean length of stay **and ** no. of beds **or** no. of beds **or** divided by number of beds? Perhaps inserting a formal equation might make this easier to interpret?
- Can you comment on why you think mortality went up? It would be interesting to know the adjusted mortality (eg for APACHE?).
- Did transfer of medical staff and equipment to COVID ICUs take place and thus limit ability to admit to non-COVID ICUs?

**Minor points:**

- In the equation stated under variables in the methods, consider using the multiplication sign '×' or even '*'.
- Was Mann- Whitney U used as normality tests indicated non-parametric data?
- What does the red line signify on Figure 1. It's quite hard to tell the difference in colour compared to the brown line.
- The axes numbering on Fig2 are almost too small to read.

I would recommend following this general rule (taken from here: https://www.nejm.org/author-center/new-manuscripts) *In general, P values larger than 0.01 should be reported to two decimal places, those between 0.01 and 0.001 to three decimal places; P values smaller than 0.001 should be reported as P<0.001.*

 Results: Outcomes - typo 'morality' instead of mortality I think.

Is the work clearly and accurately presented and does it cite the current literature?

Yes

If applicable, is the statistical analysis and its interpretation appropriate?

I cannot comment. A qualified statistician is required.

Are all the source data underlying the results available to ensure full reproducibility?

Yes

Is the study design appropriate and is the work technically sound?

Yes

Are the conclusions drawn adequately supported by the results?

Yes

Are sufficient details of methods and analysis provided to allow replication by others?

Yes

Reviewer Expertise:

Intensive Care

I confirm that I have read this submission and believe that I have an appropriate level of expertise to confirm that it is of an acceptable scientific standard, however I have significant reservations, as outlined above.

# Response

**Published:** 2021-11-05

**Authors:** IRIS India

**Affiliations:** Indian Registry of IntenSive care, India

We would like to thank the reviewers for their important and insightful feedback. The feedback has helped improve the manuscript and we are grateful for this. We address their comments below. Reviewer comments are presented in italics.

***Comment:***

*Thank you for asking me to review this manuscript analysing non-COVID ICU utilisation before and during the peak COVID pandemic in India. It demonstrates a significant disruption in healthcare utilisation during this time.*

*Overall the study is well designed and well written and acknowledges its strengths and limitations.*

**Response:** Thank you.

***Major comments are:***

*Could you comment on how the time periods were assigned. Would it be better to compare equal time periods (currently 5 months versus 7 months)?*

**Response:**

 Thank you- the choice of the time periods was based on the following reasons:

 All 13 units in the registry were fully onboarded and collecting data only by October 2019- hence the decision to use this as the start of the pre-pandemic period. While the first case of COVID-19 was reported in India on January 30 ^th^2020, the surge in cases only started in the 3 ^rd^ week of March with the imposition of the lockdown on 24 ^th^ March 2020. The first wave of the pandemic continued up until September/October 2020. Hence this was chosen as the ‘pandemic period’. We acknowledge that these separations can be artificial,  but had to pick a time point for the purposes of the analysis. However, we don’t think that the slightly unequal time periods ( 5 vs. 7 months) had a material impact on the magnitude or direction of the results, especially because the results are reported per week within each period, and not by period.

*Can you elaborate on how the unit occupancy equation definition was decided on? Did it include mean length of stay **and ** no. of beds **or** no. of beds **or ** divided by number of beds? Perhaps inserting a formal equation might make this easier to interpret?*

**Response:**

 Thank you. We have clarified ( Page number 13, Section- ‘Methods’, sub-section ‘Variables’ , Paragraph 2):

 “Unit occupancy was defined in the registry as (weekly number of admissions × mean weekly length of stay)/(number of beds available × 7). Unit turnover was defined in the registry as (weekly number of discharges/(number of beds available × 7 *)* and bed availability was defined as (weekly number of admissions × mean length of stay)-(bed capacity × 7)”.

*Can you comment on why you think mortality went up? It would be interesting to know the adjusted mortality (eg for APACHE?).*

**Response: **Thank you. Our hypotheses:

- This represents a real change due to patients presenting late (and sicker) either due to the fear of contracting COVID-19 when they come in contact with the healthcare system or due to pandemic imposed restrictions on access to healthcare facilities (a combination of lockdown and re-prioritization of healthcare beds and services for COVID-19). We have provided some references that support these theories in the ‘Discussion’ section.
- This represents a chance finding and may be a reflection of secular trends. We tried to limit this by extending the time periods of the analysis, but cannot be certain that we have fully excluded such effects.
- We have not performed an adjusted analysis as we are not confident that all information on confounders is available and/or are measured.

**We have now added a paragraph in the ‘Discussion’ section on these possible hypotheses.**

******

*Did transfer of medical staff and equipment to COVID ICUs take place and thus limit ability to admit to non-COVID ICUs?*

**Response: **Thank you- this is an interesting hypothesis, and we don’t have data to either confirm or refute this. At all participating sites, designated areas of the hospital were assigned for COVID-19 care. While hospitals did indeed increase their manpower and infrastructure capacity (by either procuring new equipment, hiring new personnel or redirecting resources from areas such as operating rooms and non- COVID wards), we don’t have data to substantiate whether this limited their capacity to admit non-COVID patients. It would indeed be interesting to explore in a future piece of work.

***Minor points:***

In the equation stated under variables in the methods, consider using the multiplication sign '×' or even '*'.

**Response**: Done

*Was Mann- Whitney U used as normality tests indicated non-parametric data?*

**Response**: Yes- we have stated this under section ‘methods’, sub-section ‘analysis’

*What does the red line signify on Figure 1. It's quite hard to tell the difference in colour compared to the brown line.*

**Response: **The red/brown lines represent the period of intense lockdown in India. We apologize that it is not clear. We have now changed this to orange for clarity.

*The axes numbering on Fig2 are almost too small to read.*

**Response**: We have now rectified this.

*I would recommend following this general rule (taken from here: https://www.nejm.org/author-center/new-manuscripts) In general, P values larger than 0.01 should be reported to two decimal places, those between 0.01 and 0.001 to three decimal places; P values smaller than 0.001 should be reported as P<0.001.*

**Response**: Thank you- we have now made the changes.

*Results: Outcomes - typo 'morality' instead of mortality I think.*

**Response**: We have changed this- thank you.

# Peer review: Reviewer response for version 1

**DOI:** 10.21956/wellcomeopenres.18709.r44617

**Published:** 2021-07-06

**Authors:** Saptharishi Lalgudi Ganesan

**Affiliations:** 1 Department of Paediatrics, Schulich School of Medicine and Dentistry, Western University, London, ON, Canada; 2 Children's Hospital of Western Ontario, London Health Sciences Center, London, ON, Canada; 3 Brain & Mind Institute, Western University, London, ON, Canada

The pandemic has disrupted the healthcare system in a multitude of ways and caused a lot of collateral impact. The authors explore the utility of critical care registries in evaluating service delivery, change in case mix or outcomes and describing the impact of the pandemic on critical care service provision overall. Using a 13 ICU registry, the authors have explored ICU occupancy during 3 time periods: pre-pandemic, pandemic with intense lockdown and the remainder of the pandemic. 

 This study captures the impact of COVID-19 on non-COVID critical care services well showing a significant reduction in the median number of admissions to the ICUs during the pandemic period as compared to the pre-pandemic period with the reduction being most prominent during the intense lockdown phase. While the median severity of illness was higher, the ICUs did not see an increase in the proportion of patients needing mechanical ventilation or cardio-vascular support at admission. Unadjusted ICU mortality was higher during the pandemic period, but the median length of stay was similar.

 The authors must be congratulated for taking this on as such studies provide valuable information regarding the collateral impact of the pandemic on critical care services and allow policy makers/leaders to plan resource allocation. The methods utilized for the purpose are appropriate. I would, however, encourage the authors to consider employing an interrupted time series approach to gain a deeper understanding of the data trends.

 As acknowledged in the limitations, having data regarding COVID and non-COVID critical care would have been valuable. In its current form, there is little information on the actual diagnoses that patients were admitted with. It would be helpful to evaluate differences if any in the case mix in a more granular manner. Also, given the scale, severity and impact of the second COVID wave in India, it would be helpful to have that time period included in this or the subsequent manuscript. We may be surprised by what we find and the results could be completely different given that healthcare services in most regions were completely overwhelmed or bursting at the seams based on media reports. 

 Overall, this work is important and efforts should be made to gather and analyze health care utilization data in all regions of India and other parts of the world so that we can be better prepared for future pandemics. As everyone now recognizes, when it comes to pandemics, it is not a matter of 'if' but 'when'.

Is the work clearly and accurately presented and does it cite the current literature?

Yes

If applicable, is the statistical analysis and its interpretation appropriate?

Yes

Are all the source data underlying the results available to ensure full reproducibility?

Yes

Is the study design appropriate and is the work technically sound?

Yes

Are the conclusions drawn adequately supported by the results?

Yes

Are sufficient details of methods and analysis provided to allow replication by others?

Yes

Reviewer Expertise:

Critical Care, Global Health, Clinical epidemiology, Neurocritical Care, Child Health

I confirm that I have read this submission and believe that I have an appropriate level of expertise to confirm that it is of an acceptable scientific standard, however I have significant reservations, as outlined above.

# Response

**Published:** 2021-11-05

**Authors:** IRIS India

**Affiliations:** Indian Registry of IntenSive care, India

We would like to thank the reviewers for their important and insightful feedback. The feedback has helped improve the manuscript and we are grateful for this. We address their comments below. Reviewer comments are presented in italics.

**Comment:** *The pandemic has disrupted the healthcare system in a multitude of ways and caused a lot of collateral impact. The authors explore the utility of critical care registries in evaluating service delivery, change in case mix or outcomes and describing the impact of the pandemic on critical care service provision overall. Using a 13 ICU registry, the authors have explored ICU occupancy during 3 time periods: pre-pandemic, pandemic with intense lockdown and the remainder of the pandemic.*

*This study captures the impact of COVID-19 on non-COVID critical care services well showing a significant reduction in the median number of admissions to the ICUs during the pandemic period as compared to the pre-pandemic period with the reduction being most prominent during the intense lockdown phase. While the median severity of illness was higher, the ICUs did not see an increase in the proportion of patients needing mechanical ventilation or cardio-vascular support at admission. Unadjusted ICU mortality was higher during the pandemic period, but the median length of stay was similar.*

*The authors must be congratulated for taking this on as such studies provide valuable information regarding the collateral impact of the pandemic on critical care services and allow policy makers/leaders to plan resource allocation. The methods utilized for the purpose are appropriate. I would, however, encourage the authors to consider employing an interrupted time series approach to gain a deeper understanding of the data trends.*

**Response**: Thank you! Regarding the suggestion for use of interrupted time series (ITS), we agree in principle that this approach would add rigour. We did not employ this technique as the assumptions required for ITS (linearity etc.) are not met in our dataset. For future analyses and as the registry grows, we will certainly consider this approach. Thank you.

 We have now added a sentence under the ‘analysis’ sub-section of the ‘methods’ to explain why an ITS approach was not taken.

**Comment:** *As acknowledged in the limitations, having data regarding COVID and non-COVID critical care would have been valuable. In its current form, there is little information on the actual diagnoses that patients were admitted with. It would be helpful to evaluate differences if any in the case mix in a more granular manner. Also, given the scale, severity and impact of the second COVID wave in India, it would be helpful to have that time period included in this or the subsequent manuscript. We may be surprised by what we find and the results could be completely different given that healthcare services in most regions were completely overwhelmed or bursting at the seams based on media reports.* 

**Response:** We agree that additional granular information on case-mix would provide crucial insights. However, the registry is currently set up to capture broad diagnostic categories and hence we were unable to provide this information. We will also consider the suggestion of the reviewer to include data from the ‘second’ wave in India in a future manuscript. We have now added this to the section on ‘limitations’.

**Comment:** *Overall, this work is important and efforts should be made to gather and analyze health care utilization data in all regions of India and other parts of the world so that we can be better prepared for future pandemics. As everyone now recognizes, when it comes to pandemics, it is not a matter of 'if' but 'when'.*

**Response**: Thank you once again for the feedback.
